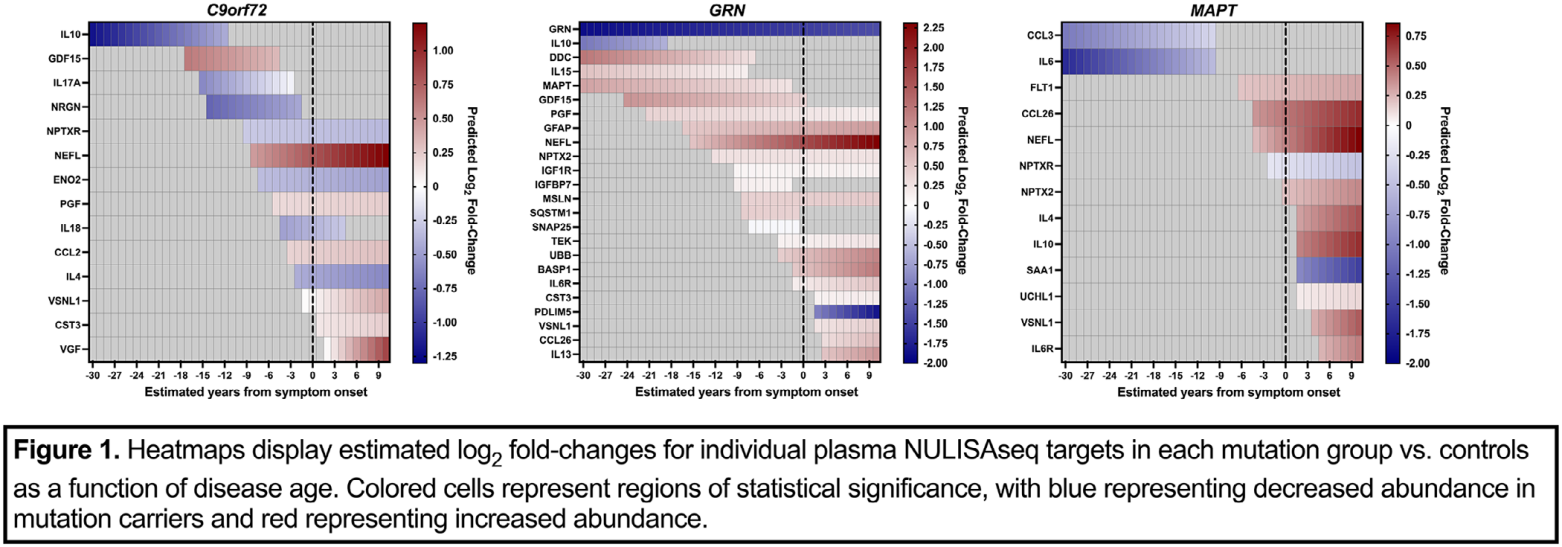# Targeted proteomics via NULISAseq identifies blood‐detectable markers of disease progression in familial frontotemporal lobar degeneration

**DOI:** 10.1002/alz70856_098644

**Published:** 2025-12-24

**Authors:** Rowan Saloner, Joshua Downer, Argentina Lario Lago, Julia D Webb, Hilary W. Heuer, Leah K. Forsberg, Julio C. Rojas, Lawren VandeVrede, Peter A. Ljubenkov, Tania F Gendron, Leonard Petrucelli, Brad F Boeve, Howard J. Rosen, Adam M. Staffaroni, Adam L. Boxer

**Affiliations:** ^1^ Memory and Aging Center, UCSF Weill Institute for Neurosciences, University of California, San Francisco, San Francisco, CA, USA; ^2^ University of California, San Francisco, San Francisco, CA, USA; ^3^ Memory and Aging Center, Weill Institute for Neurosciences, University of California San Francisco, San Francisco, CA, USA; ^4^ Memory and Aging Center, Weill Institute for Neurosciences, University of California, San Francisco, San Francisco, CA, USA; ^5^ Mayo Clinic, Rochester, MN, USA; ^6^ Memory and Aging Center, UCSF Weill Institute for Neurosciences, University of California San Francisco, San Francisco, CA, USA; ^7^ Mayo Clinic, Jacksonville, FL, USA; ^8^ Memory and Aging Center, Department of Neurology, Weill Institute for Neurosciences, University of California, San Francisco, San Francisco, CA, USA

## Abstract

**Background:**

Recent technological advances have enhanced the precision of blood‐based multiplex proteomics and their application in Alzheimer's disease. However, these approaches remain underutilized in frontotemporal lobar degeneration (FTLD). Familial FTLD cohorts offer a unique opportunity to study presymptomatic periods of disease that cannot be studied in sporadic cases. We leveraged NUcleic acid Linked Immuno‐Sandwich Assay (NULISAseq) to identify blood‐detectable proteins that change prior to symptom onset in familial FTLD.

**Method:**

120 FTLD mutation carriers (40 *C9orf72*, 40 *GRN*, 40 *MAPT*) and 40 age and sex‐matched noncarrier controls from the ALLFTD Consortium completed blood draws and clinical assessment. Targeted plasma proteomics was performed via NUcleic acid Linked Immuno‐Sandwich Assay (NULISAseq; 132 proteins). Disease age estimates measuring predicted symptom onset were derived from a validated model incorporating FTLD gene‐specific clinical, neuroimaging, and biomarker data. Mass linear regression models identified disease age ‘thresholds’ where individual proteins significantly diverged between each mutation carrier group and controls. NULISAseq targets were validated against orthogonal assays, specifically Simoa (plasma) and SomaScan (cerebrospinal fluid [CSF]).

**Result:**

Compared to controls, 14 plasma proteins significantly diverged (*p* <.05) in *C9orf72*, 24 in *GRN*, and 13 in *MAPT*. Beyond NEFL, 10 additional proteins significantly diverged in more than one mutation group, including markers of synaptic function (NPTX2, NPTXR) and vascular integrity (PGF) that deviated prior to estimated symptom onset (disease age range: ‐30 to 0). Proteins with gene‐specific signals included GFAP (increased in *GRN* at disease age ‐17), ENO2 (decreased in *C9orf72* at disease age ‐7), and FLT1 (increased in *MAPT* at disease age ‐6). Plasma NULISA‐based NEFL and GFAP showed strong concordance when measured via plasma Simoa (*r*s>0.88) and were among 38 targets with significant concordance when measured via CSF SomaScan (*r* range: 0.16 to 0.75, *p*s<.05).

**Conclusion:**

Alterations in plasma protein abundance are measurable in familial FTLD prior to predicted symptom onset. The validity and brain‐based relevance of blood‐detectable NULISA targets is bolstered by the observed convergence with plasma Simoa and CSF SomaScan. Replication of top hits in larger familial and sporadic FTLD sample sizes is warranted to determine generalizability and suitability for clinical trials.